# Supplementary material for: Awake ripples enhance emotional memory encoding in the human brain
Source: Nat Commun. 2024 Jan 3;15:215. doi: 10.1038/s41467-023-44295-8 (PMC10764865; doi:10.1038/s41467-023-44295-8)
Supplement: Supplementary file 2 — Supplementary Information [file 41467_2023_44295_MOESM2_ESM.pdf]

Supplementary Materials for

Awake ripples enhance emotional memory encoding in the human brain

Haoxin Zhang*#, Ivan Skelin#, Shiting Ma, Michelle Paff, Lilit Mnatsakanyan, Michael A. Yassa, Robert T. Knight & Jack J. Lin*

*Corresponding author. Email: haoxinz1@uci.edu; jajlin@ucdavis.edu

# Equal contribution

This PDF file includes:

Supplementary Fig. 1 to 17

Supplementary Table 1 to 3

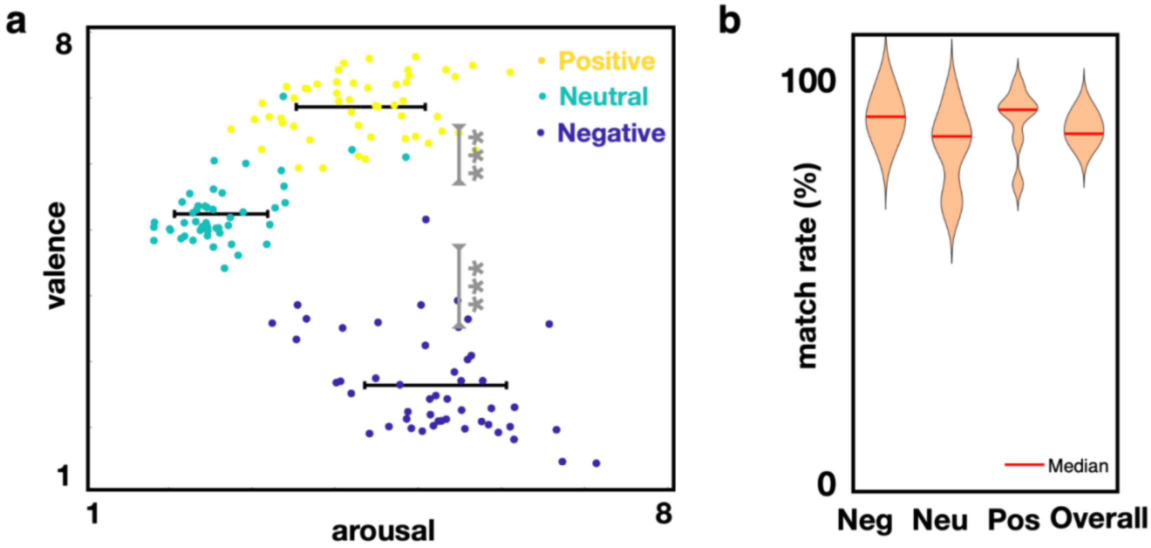

Supplementary Fig. 1. **Relation between the stimulus emotional valence and arousal. a**, Positive and negative valenced stimuli (based on healthy participant ratings) are associated with higher stimulus-induced arousal, relative to neutral valence stimuli (***p<0.001, Wilcoxon rank-sum test). Box and bar indicate mean ± SD. **b**, Stimuli valence ratings of study participants are highly similar to the healthy population (match rate = 85.3 ± 1.3%).

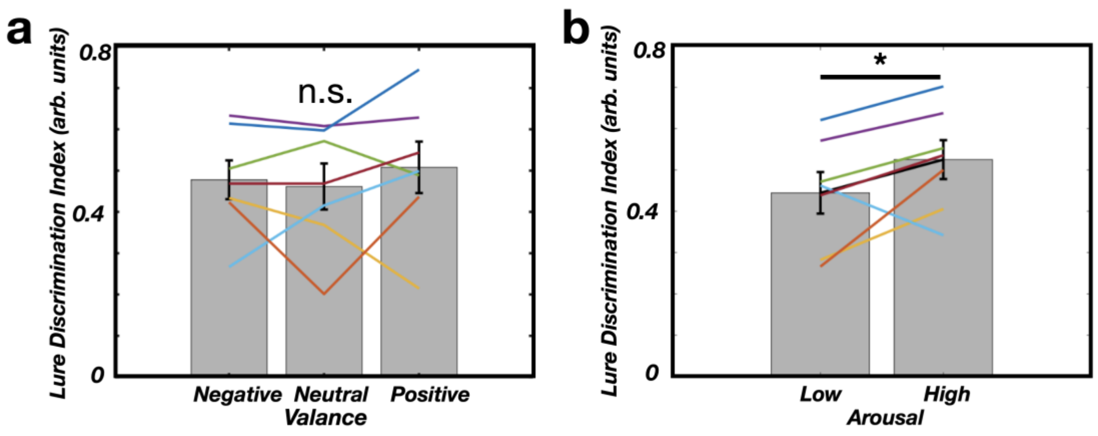

Supplementary Fig. 2. Lure discrimination index (LDI) is not significantly associated with the stimulus valence, while it is significantly higher for the high-arousal stimuli reflecting the tendency for high-arousal stimuli to be classified as ‘New’. **a,** Valence: p = 0.396, F(2, 18) = 0.980; one-way ANOVA; **b**, Arousal: p = 0.043, t(6) = -2.058, one-tailed paired t-test. The data from individual participants are color-coded. Box and bar indicate mean ± SEM.

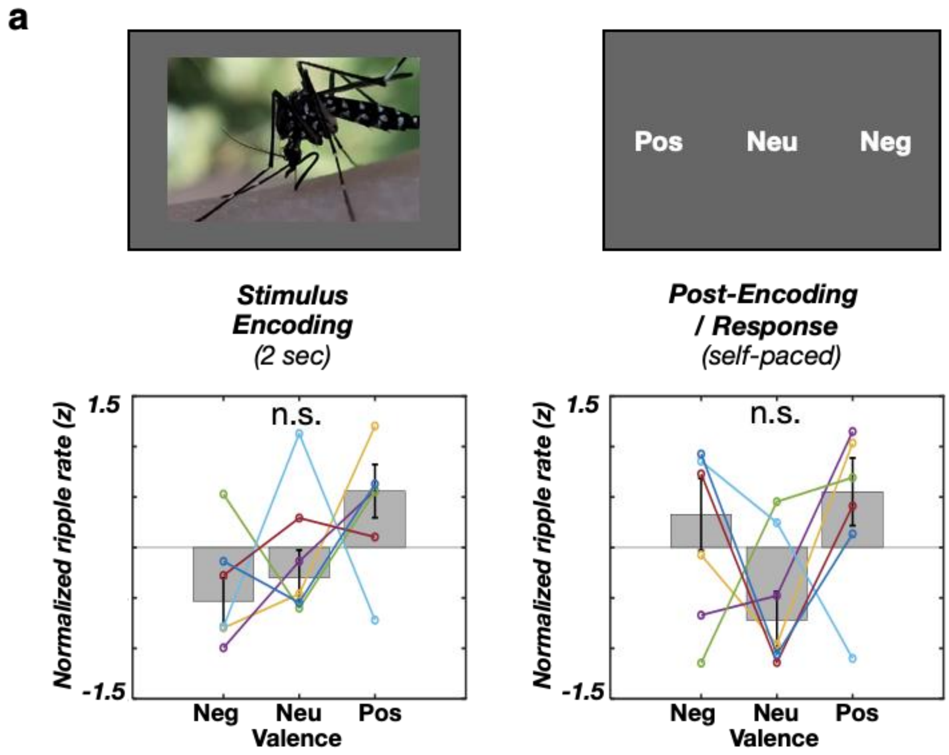

Supplementary Fig. 3. Stimulus valence is not significantly associated with ripple rate during the encoding or post-encoding epochs. Stimulus encoding phase: $F(2, 15) = 2.44$, $p = 0.121$; Post-encoding: $F(2, 15) = 1.88$, $p = 0.187$, One-way ANOVA). The data from individual participants are color-coded. Box and bar indicate mean ± SEM.

a

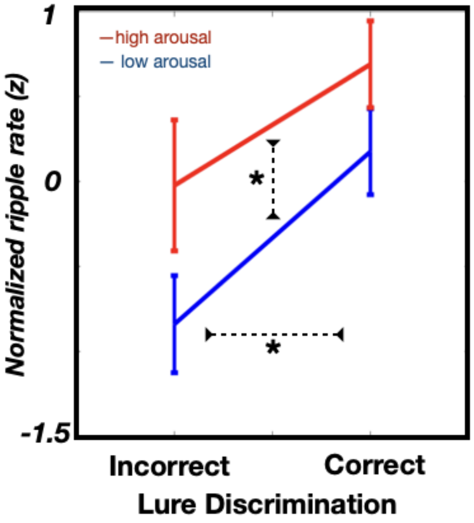

Supplementary Fig. 4. Stimulus-induced arousal and later correct Lure discrimination independently associated with the post-encoding ripple rate. The post-encoding ripple rate was significantly associated with both stimulus-induced arousal ($p=0.038$, $F(1,20) = 4.93$) and later correct Lure discrimination ($p = 0.009$, $F(1,20) = 8.32$), without significant interaction ($p = 0.619$, $F(1,20) = 0.26$; two-way ANOVA). Box and bar indicate mean ± SEM.

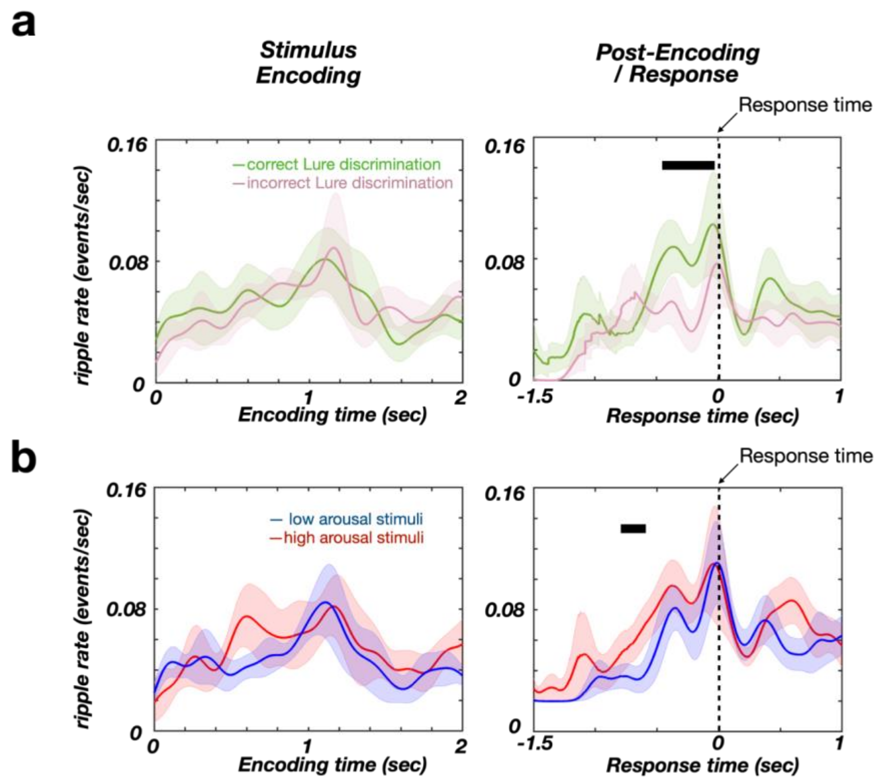

Supplementary Fig. 5. The time-resolved association between ripple rate and stimulus arousal/correct Lure discrimination across the task epochs. a, During stimulus encoding, there was no significant ripple rate difference between the correctly and incorrectly discriminated Lure stimuli (top; $p > 0.05$, non-parametric cluster-based permutation test) or low-arousal and high-arousal Lure stimuli (bottom; $p > 0.05$, non-parametric cluster-based permutation test). **b,** During post-encoding, ripple rates were significantly higher for correctly discriminated, relative to incorrectly discriminated Lure stimuli (top, $p = 0.005$, -400 to -50 msec relative to response time), and for high-arousal, relative to low-arousal Lure stimuli (bottom, $p = 0.035$, -780 to -600 msec relative to response time, non-parametric cluster-based permutation test). Line and shaded areas represent the mean ± SEM.

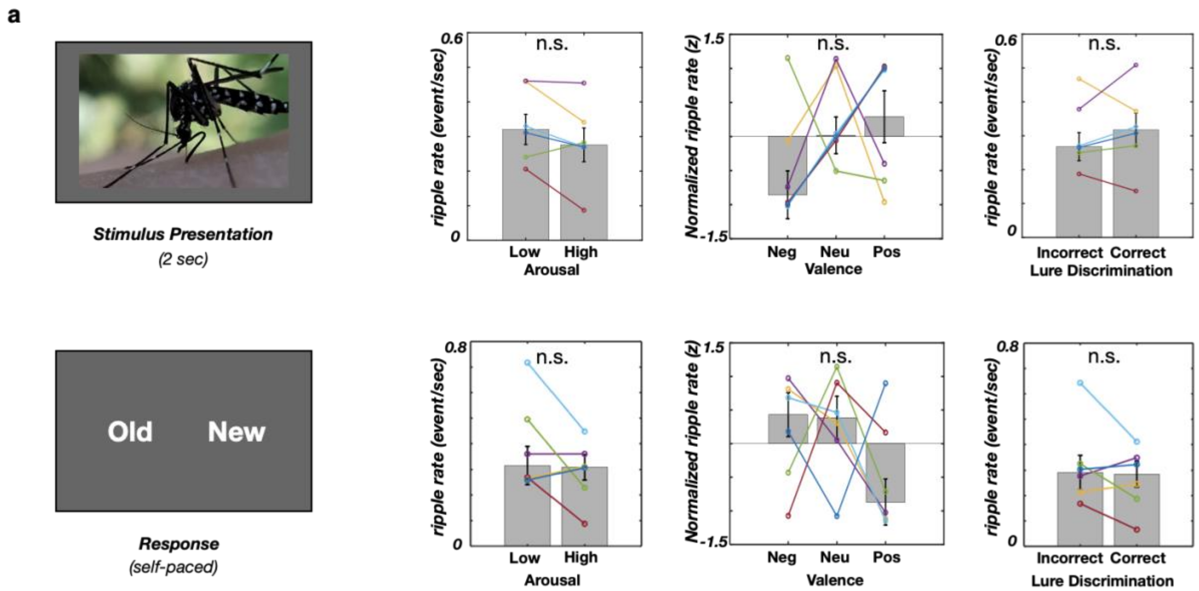

Supplementary Fig. 6. The ripple *rate* during retrieval task stage is not associated with stimulus-induced arousal, valence or correct Lure discrimination. Arousal: Stimulus presentation (top row), p = 0.116, z = 1.57; Response (bottom row), p = 0.*249*, z = *1.15*, Wilcoxon signed-rank test. **Valence**: Stimulus presentation, p = 0.*298*, F(2, 15) = *1.32*; Response, p = 0.*220*, F(2, 15) = *1.68*, One-way ANOVA). **Correct Lure discrimination**: Stimulus presentation: p = 0.600, z = -0.52; Response: p = 0.*345*, z = *0.94*, Wilcoxon signed-rank test). Box and bar indicate mean ± SEM.

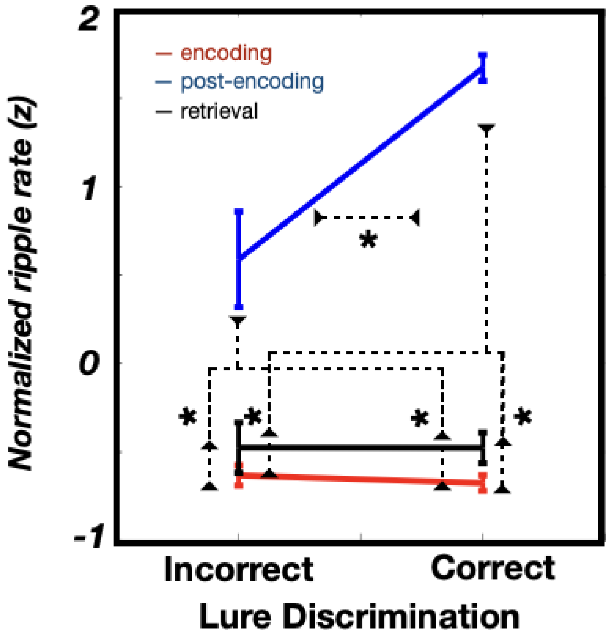

Supplementary Fig. 7. The association between ripples and correct Lure discrimination is selective for post-encoding epoch. Only the post-encoding ripple rates are associated with correct Lure discrimination (significant epoch x Lure discrimination interaction effect, $p < 0.001$, $F(2,30) = 10.97$, two-way ANOVA). Post-hoc analysis has shown the significantly higher ripple rates during post-encoding epoch for the correctly discriminated Lure stimuli (post-encoding: $p < 0.001$, $M = -1.70$ with 95% CI = [-1.08 , -0.47]), while there was no significant difference during the encoding or retrieval epochs (p's > 0.05; post-hoc tests performed using multcompare.m function in Matlab). Box and bar indicate mean ± SEM.

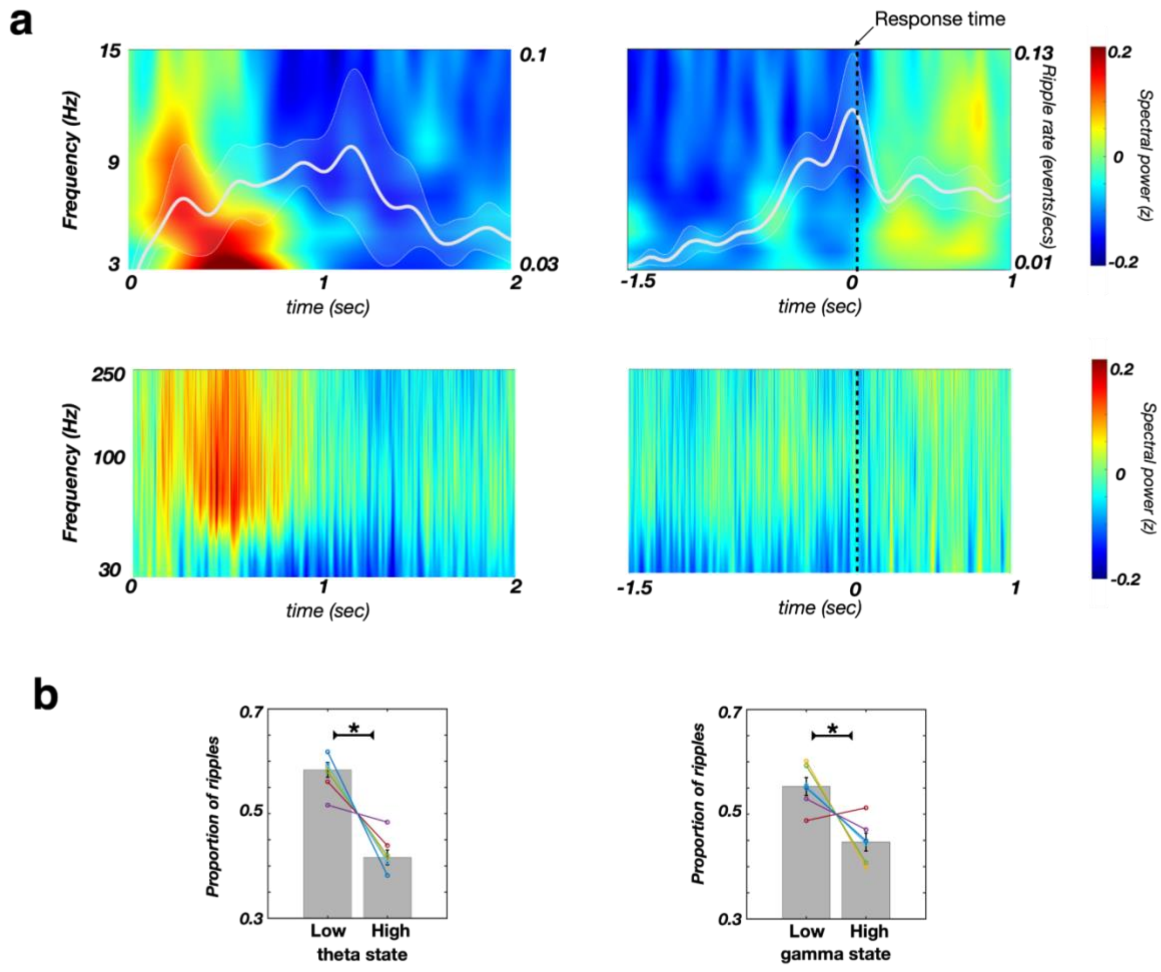

Supplementary Fig. 8. Ripples occur predominantly outside of high theta or broadband gamma periods. a, Low frequency (top, color) and high frequency spectrogram (bottom, color), and ripple rate (white line) during the stimulus encoding (left) and post-encoding (right, response-locked) periods. **b,** Ripple proportion is lower during the high theta state (top, $p = 0.017$, $z = 2.1$, one-tailed Wilcoxon signed-rank test), or during high gamma state (bottom, $p = 0.028$, $z = 1.9$, one-tailed Wilcoxon signed-rank test). Box and bar indicate mean ± SEM. Theta/gamma state classification was based on the power median split (for details, see 'Dual state analysis').

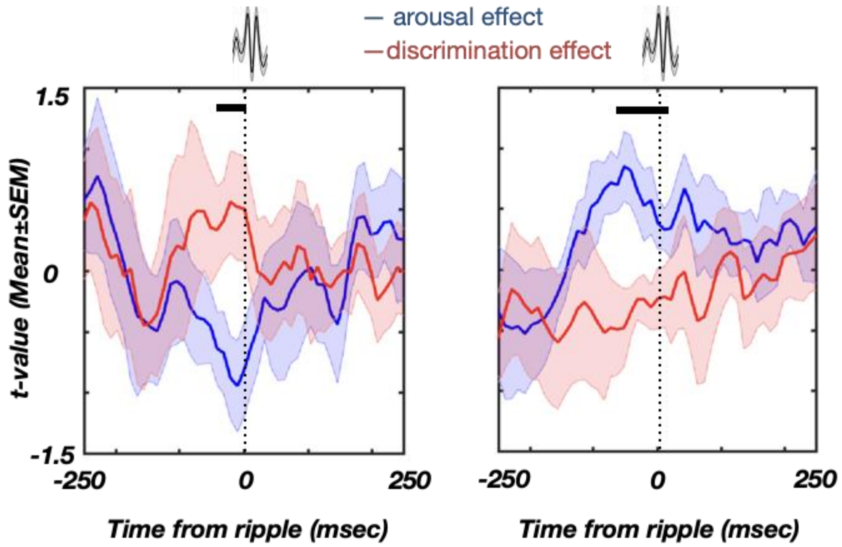

Supplementary Fig. 9. Double-dissociation between the post-encoding ripple-locked stimulus similarity in hippocampus and amygdala. Left: The association between the stimulus arousal and post-encoding ripple-locked stimulus similarity was stronger in the amygdala (-42 msec to 0 msec relative to ripple peak, p = 0.034, non-parametric cluster-based permutation test). Right: The association between the later correct Lure discrimination and post-encoding ripple-locked stimulus similarity was stronger in the hippocampus (-83 msec to 10 msec relative to ripple peak, p = 0.047, non-parametric cluster-based permutation test). The line and shaded areas represent the mean ± SEM of the individual participant t-values, respectively.

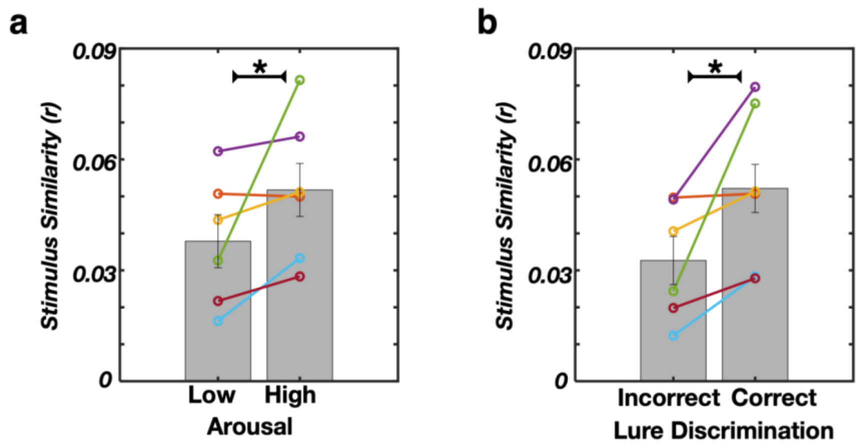

Supplementary Fig. 10. Post-encoding ripple-locked stimulus similarity (amygdala and hippocampus combined) is increased for high stimulus-induced arousal and correctly discriminated stimuli. a, Arousal: *p = 0.046, z(5) = -1.991, Wilcoxon signed-rank test. **b**, Correct Lure discrimination: *p = 0.028, z(5) = -2.201, Wilcoxon signed-rank test). Data from individual participants is color-coded. Box and bar indicate mean ± SEM.

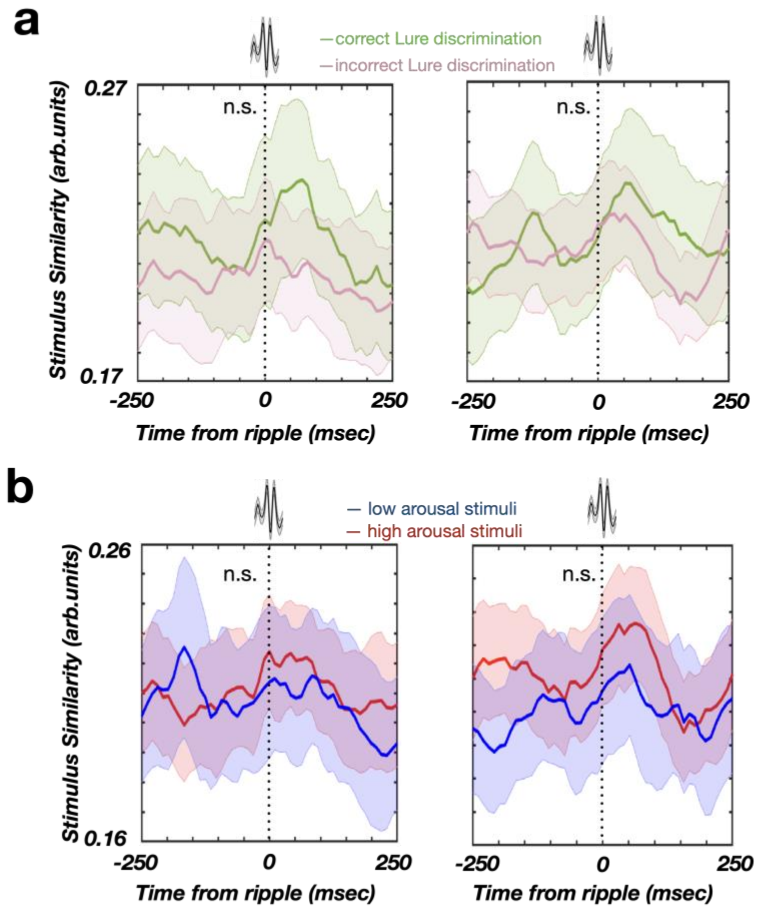

Supplementary Fig. 11. Surrogate event-locked similarity on the trials not containing post-encoding ripples. Post-encoding similarity in the amygdala (left) and hippocampus (right) was not associated with the stimulus arousal (bottom) or later correct Lure discrimination (top). Non-parametric cluster-based permutation test, p's > 0.05. Line and shaded areas represent the mean ± SEM.

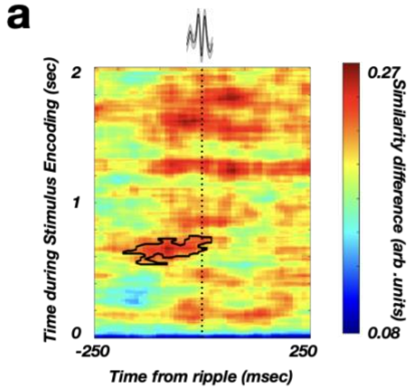

Supplementary Fig. 12. The representational similarity map showing the stimulus specificity of the post-encoding ripple representation in the hippocampus. The temporal cluster of significant stimulus-specific similarity (-190 - 20 msec, relative to ripple peak and ~500-750 msec of encoding time) is encircled in black (non-parametric cluster-based permutation test; n = 1000 permutations, $p < 0.05$).

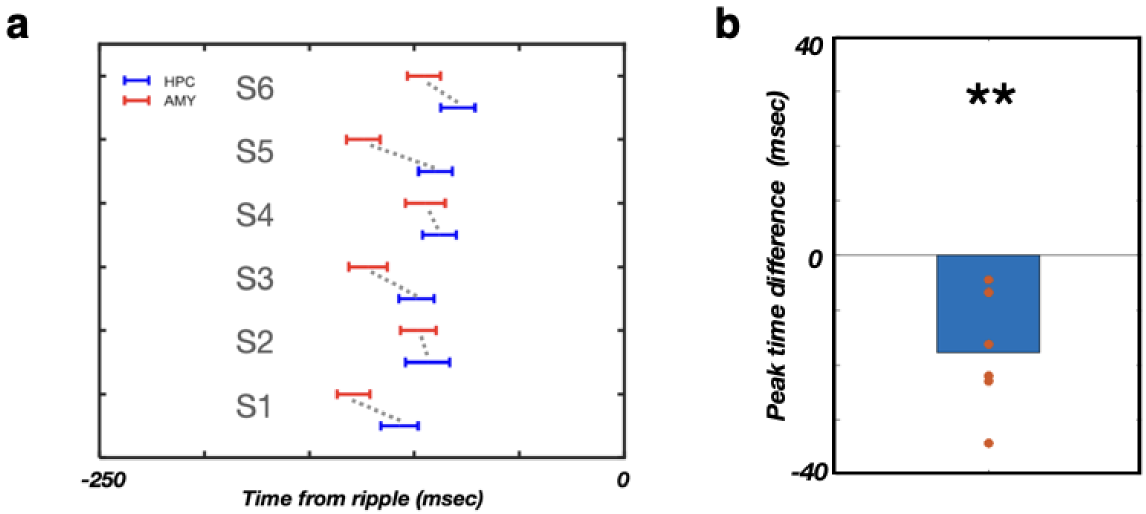

Supplementary Fig. 13. Ripple-locked similarity occurs in the amygdala, prior to hippocampus. a, Stimulus similarity during post-encoding ripple windows peaks earlier in the amygdala (red), relative to hippocampus (blue). The data points represent individual participant mean ± SD. The earlier amygdala similarity peak is consistent across the individual participants. **b**, The difference in ripple-locked peak similarity timing (amygdala - hippocampus). Negative values denote the earlier similarity peak in the amygdala (-18 ± 11 msec, mean ± SEM; $p = 0.006$, $t(5) = -3.89$; one-tail paired t-test).**$p < 0.01$

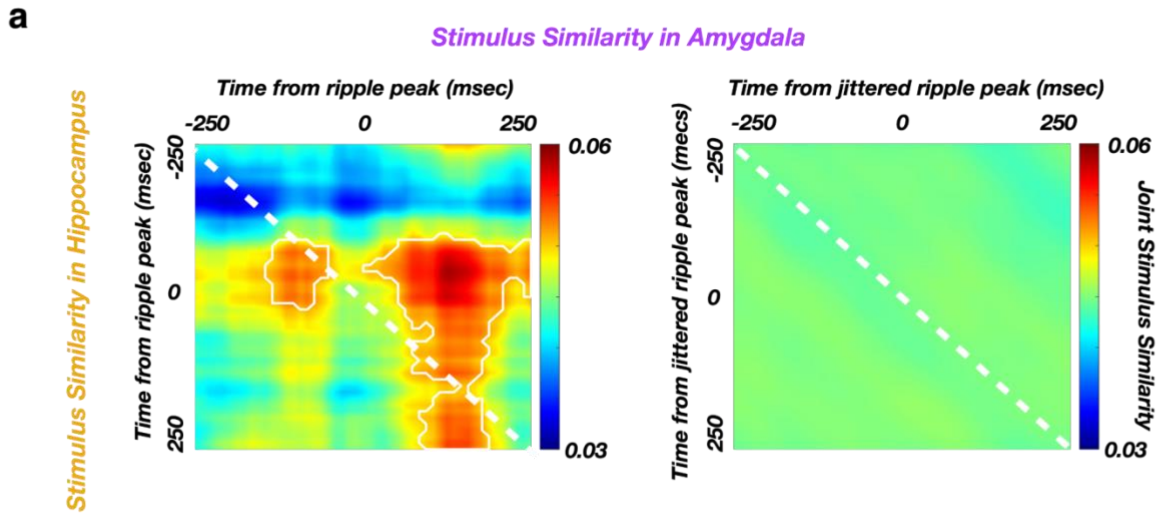

Supplementary Fig. 14. Joint cross-structure post-encoding stimulus similarity occurs selectively during ripple time windows. a, Average joint cross-structure post-encoding stimulus similarity (hippocampus and amygdala) relative to ripple peak times (left) and relative to jittered ripple peak times (right). The white line encircles the periods of significant joint cross-structure post-encoding stimulus similarity (Fig. 3d). The color scale represents the Spearman correlation between the encoding stimulus presentation and post-encoding ripple windows. The absence of significant joint cross-structure post-encoding stimulus similarity following the jittering of ripple peak times (right) reveals the specificity of cross-structure stimulus similarity to ripple windows.

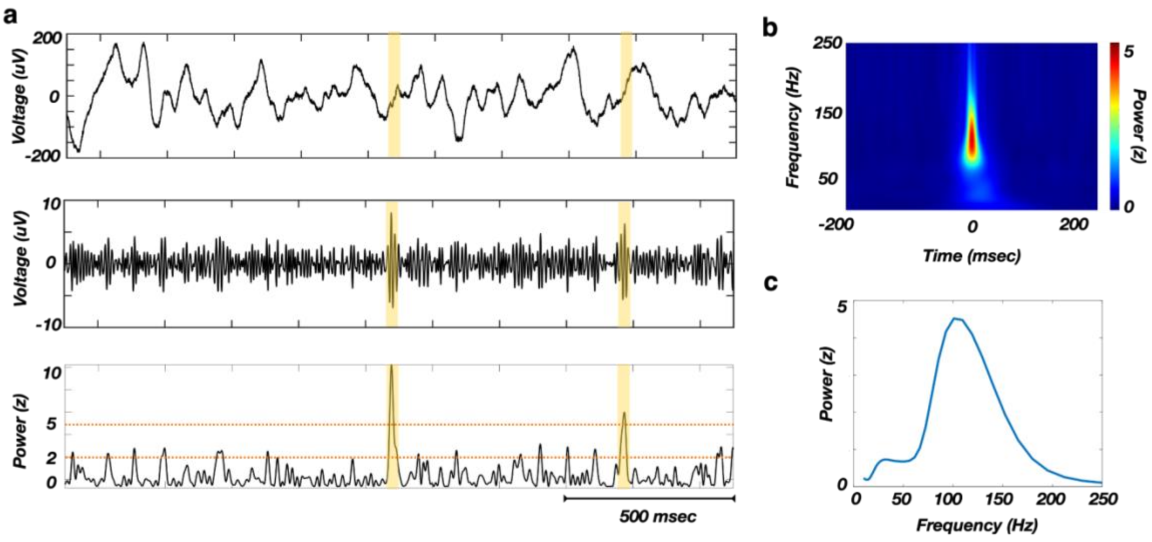

Supplementary Fig. 15. Awake ripple detection. a, Examples of several detected ripples (yellow highlights), showing the raw trace (top), filtered trace (80 - 150 Hz range, middle) and z-scored envelope of filtered trace (bottom). Detection is based on double-threshold (orange dashed lines) crossing of z-scored power (80-150 Hz) for the period of 20-100 msec. **b**, Z-scored power spectral density of average detected ripple. **c**, Z-scored power during ripple windows shows a bump in the 80-150 Hz range. This suggests that the ripples are not detected during signal artifact periods, which would reflect as a broadband power increase. In addition, detected ripples are not detected during non-specific increase in broadband gamma power or pathological high-frequency oscillations (> 200 Hz).

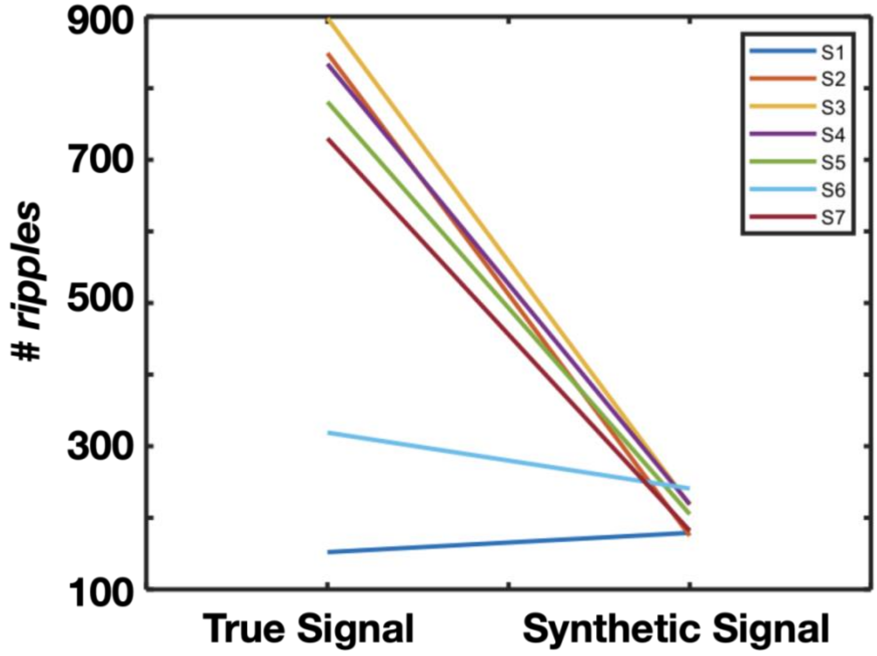

Supplementary Fig. 16. Ripple detection in the hippocampal signal and synthetic signal. Comparisons between the numbers of ripples detected in hippocampal channels (left) and number of events detected in participant-specific synthetic signals of the same spectral characteristics (right). In all the participants included in ripple analysis (participants 2-7) ripple detection in hippocampal signal was higher than in synthetic signal. Participant 1 was excluded from the ripple-based analysis due to low ripple detection in hippocampal signal (z-score < -2, relative to distribution across the participants) and the number of detected putative ripples lower than in synthetic signals of the same spectral characteristics.

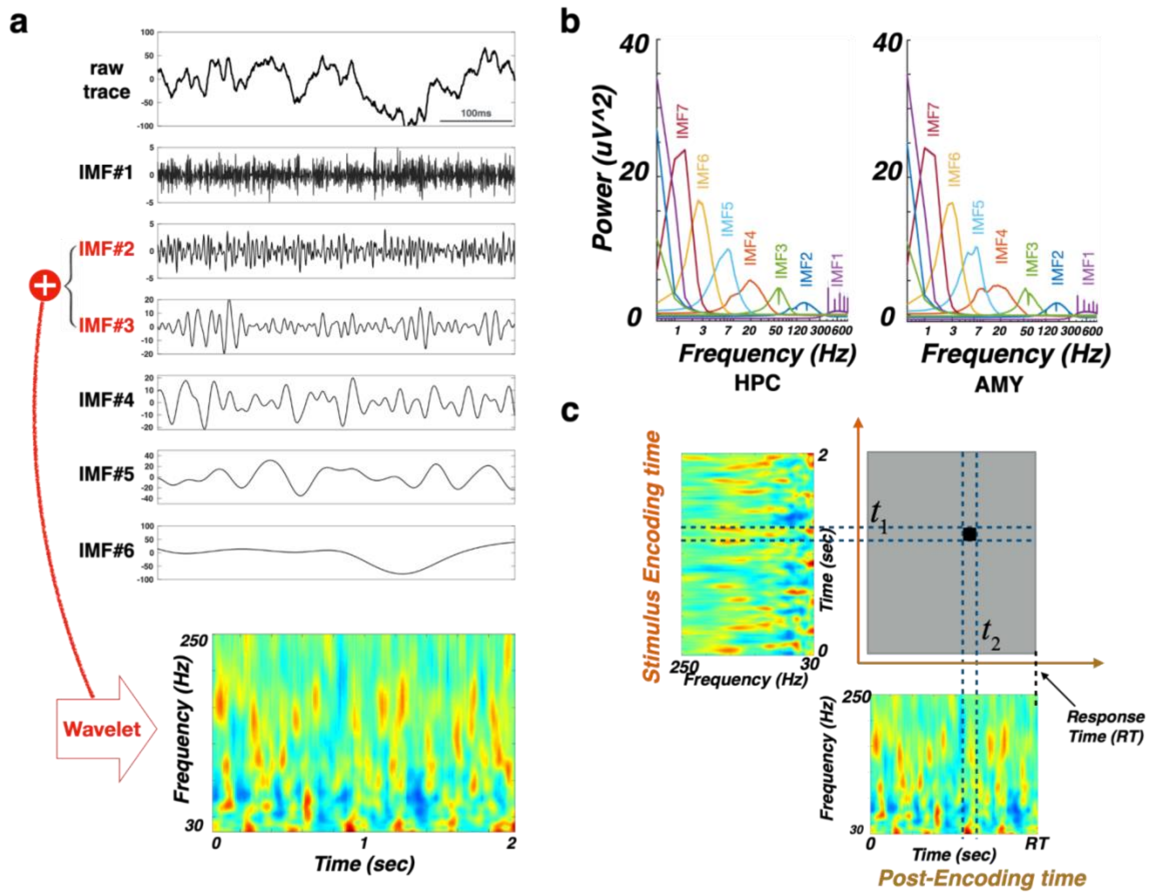

Supplementary Fig. 17. Overview of Ensemble Empirical Mode Decomposition (EEMD) and representational similarity analysis (RSA) methods. a, An example hippocampal raw iEEG trace (top) was decomposed into multiple intrinsic mode functions (IMFs; lower 6 panels). IMFs within the HFA range (IMF_2 and IMF_3) were used for HFA reconstruction. The HFA time-frequency matrix (bottom) was estimated using wavelet transformation (for details, see Time-frequency representation of the HFA). **b,** Power spectral density (mean ± SEM) of the IMFs decomposed from the hippocampal (left) and amygdala (right) electrodes. IMF spectral features were consistent across participants and structures, with mean center frequencies in delta (IMF_7), theta (IMF_6, IMF_5), alpha/beta (IMF_4), gamma (IMF_3), high-gamma bands (IMF_2), and the noise term (IMF_1). The HFA time series were estimated by summing the IMFs with center frequencies > 30 Hz (IMF_2 and IMF_3). **c**, The similarity matrix (top right) was constructed by computing the power spectrum vector (PSV) Spearman's correlations for each combination of stimulus encoding (top left) and post-encoding (bottom right) time bins.

Supplementary Table 1. Demographic information for the study participants.

Participant	Gender	Age Range
S1	M	20 - 30
S2	F	50 - 60
S3	M	20 - 30
S4	F	50 - 60
S5	M	20 - 30
S6	M	20 - 30
S7	F	20 - 30

Supplementary Table 2. Statistics summary of two-sided logistic linear mixed effect model with three main effects (Valence, Arousal, Similarity) on correct Lure discrimination and all the interactions. *p<0.05.

	beta	t-values	df	p-value
**Intercept***	-0.386	-17.406	448	$3.31*10^{-52}$
**Valence**	0.036	1.020	448	0.308
**Arousal***	0.483	15.782	448	$6.15*10^{-45}$
**Similarity***	0.736	50.562	448	$2.99*10^{-187}$
**Valence x Arousal**	$-4.1 * 10^{-12}$	-2.7 * 10-5	448	0.999
**Valence x Similarity**	$1.6 * 10^{-12}$	-4.9 * 10-5	448	0.999
**Arousal x Similarity***	0.516	10.327	448	$1.44*10^{-22}$
**Valence x Arousal x Similarity***	-0.442	-17.149	448	$4.81*10^{-51}$

Supplementary Table 3. Center frequencies of the IMFs in the hippocampus and amygdala.

	Hippocampus	Amygdala
IMF#1	420 Hz	420 Hz
IMF#2	159 Hz	184 Hz
IMF#3	57.5 Hz	53 Hz
IMF#4	20 Hz	18 Hz
IMF#5	8 Hz	7.5 Hz
IMF#6	2.5 Hz	3 Hz
IMF#7	1.5 Hz	1 Hz
IMF#8	0.5 Hz	0.5 Hz
IMF#9	< 0.5 Hz	< 0.5 Hz
IMF#10	< 0.5 Hz	< 0.5 Hz
